# The Relevance of Amyloid β-Calmodulin Complexation in Neurons and Brain Degeneration in Alzheimer’s Disease

**DOI:** 10.3390/ijms22094976

**Published:** 2021-05-07

**Authors:** Joana Poejo, Jairo Salazar, Ana M. Mata, Carlos Gutierrez-Merino

**Affiliations:** 1 Instituto de Biomarcadores de Patologías Moleculares, Universidad de Extremadura, 06006 Badajoz, Spain; joanapoejo86@gmail.com (J.P.); jairochemsalazar@gmail.com (J.S.); anam@unex.es (A.M.M.); 2 Departamento de Química, Universidad Nacional Autónoma de Nicaragua-León, León 21000, Nicaragua; 3 Departamento de Bioquímica y Biología Molecular y Genética, Facultad de Ciencias, Universidad de Extremadura, 06006 Badajoz, Spain

**Keywords:** amyloid β, calmodulin, neurons, Alzheimer’s disease, calmodulin-binding proteins, intracellular calcium dysregulation, calcium signaling, neuronal functions, brain degeneration

## Abstract

Intraneuronal amyloid β (Aβ) oligomer accumulation precedes the appearance of amyloid plaques or neurofibrillary tangles and is neurotoxic. In Alzheimer’s disease (AD)-affected brains, intraneuronal Aβ oligomers can derive from Aβ peptide production within the neuron and, also, from vicinal neurons or reactive glial cells. Calcium homeostasis dysregulation and neuronal excitability alterations are widely accepted to play a key role in Aβ neurotoxicity in AD. However, the identification of primary Aβ-target proteins, in which functional impairment initiating cytosolic calcium homeostasis dysregulation and the critical point of no return are still pending issues. The micromolar concentration of calmodulin (CaM) in neurons and its high affinity for neurotoxic Aβ peptides (dissociation constant ≈ 1 nM) highlight a novel function of CaM, i.e., the buffering of free Aβ concentrations in the low nanomolar range. In turn, the concentration of Aβ-CaM complexes within neurons will increase as a function of time after the induction of Aβ production, and free Aβ will rise sharply when accumulated Aβ exceeds all available CaM. Thus, Aβ-CaM complexation could also play a major role in neuronal calcium signaling mediated by calmodulin-binding proteins by Aβ; a point that has been overlooked until now. In this review, we address the implications of Aβ-CaM complexation in the formation of neurotoxic Aβ oligomers, in the alteration of intracellular calcium homeostasis induced by Aβ, and of dysregulation of the calcium-dependent neuronal activity and excitability induced by Aβ.

## 1. Intracellular Amyloid β (Aβ) Oligomers in Neuronal Cytotoxicity and Calmodulin (CaM) as a Major High Affinity Aβ-Binding Protein in Neurons

Intraneuronal Aβ accumulation has been shown to mediate neuronal cytotoxicity, and it has been suggested to be an early pathological biomarker for the onset of Alzheimer’s disease (AD) [1]. This is also supported by the finding that intraneuronal Aβ accumulation precedes the appearance of amyloid plaques or tangles in transgenic mice [2,3,4,5], and that it also correlates with alteration of long-term potentiation, synaptic dysfunction, and memory impairment in a triple transgenic model of AD [3,6]. The prevalent Aβ peptide found in the amyloid plaques of AD patients is Aβ(1–42) [7]. It has been shown that microinjection of Aβ(1–42) or cDNA encoding Aβ(1–42) is neurotoxic to human neurons in culture [8]. Furthermore, oligomeric species of Aβ(1–42) are tightly linked to AD pathogenesis and are likely to be the cause of neuronal damage [9,10,11]. In addition, neuronal uptake and accumulation of Aβ(1–42) aggregates correlated with metabolic inhibition [12], while extracellular Aβ, applied to hippocampal slices, seems to preferentially target synapses, leading to a decrease in the synaptic marker synaptophysin [13], as well as endocytosis of N-methyl-D-aspartate receptors (NMDAR) [14] and low-density lipoprotein receptor-related protein-1 (LRP1) [15,16]. Of note, the plasma membrane distribution and internalization of NMDARs are modulated by LRP1 [17], and it has been proposed that physical interactions with LRP1 may also mediate the functional modulation of NMDAR by LRP1 [18,19]. In addition, the binding of extracellular Aβ to other proteins, such as the α7 nicotinic cholinergic receptor, ApoE and ApoE receptors, integrins and the receptor for advanced glycation end products, has been shown to be implicated in Aβ uptake by neurons and was reviewed in [20].

Calmodulin (CaM) is a calcium buffering protein which is expressed in neurons at much higher concentrations than in non-excitable cells, reaching micromolar concentrations in neurons [21]. The levels of CaM expressed in the brain are within 4 and 15 μg/mg wet tissue, with highest content in cortical regions, striatum, hippocampus, amygdala, and substantia grisea [22]. We showed that calcium-saturated CaM binds with high affinity to Aβ(1–42) and Aβ(25–35) peptides, as demonstrated by a dissociation constant of the Aβ:Ca^2+^_4_-CaM complex close to 1 nM [23]. As a result, CaM could have a high capacity to buffer intracellular free Aβ concentrations. We have recently shown [24] that primary cultures of mature cerebellar granule neurons express 5.5 ± 0.5 ng of CaM per µg of total protein, i.e., approximately 1 μg of CaM or 56 ± 6 picomoles of CaM in a plate seeded with 2.5 × 10^6^ neurons. Additionally, we found that CaM extensively binds Aβ(1–42) dimers in cerebellar granule neurons after only 2 hours of incubation at 37 °C with micromolar concentrations of Aβ(1–42) dimers added to the extracellular medium, which allowed to calculate that the CaM present in these neurons can bind up to around 120 picomoles of Aβ/2.5 × 10^6^ neurons [24]. This latter result leads to the complexation of the micromolar intracellular CaM concentration, considering the size and the internal volume of mature cerebellar granule neurons. Other proteins known to bind Aβ peptides with dissociation constants close to 1 nM, i.e., with an affinity similar to that of CaM, are cellular prion protein [25] and glycogen synthase kinase 3α [26]. However, the expression level of these proteins in neurons is several orders of magnitude lower than that of CaM. Therefore, in neurons, CaM seems to be a major sink for neurotoxic intracellular Aβ peptides, and this, in turn, suggests that CaM could play a key role in protecting against an increase of free intracellular Aβ concentrations above 1–2 nM. Based on this, it can be expected that down-regulation of the expression of CaM should make neurons more prone to Aβ-induced neurotoxicity, because they will suffer a stronger rise in the free intracellular concentration of Aβ peptides upon β-secretase activation or extracellular Aβ uptake. Of note, a decrease in the CaM expression level in brains affected by AD has been reported [27].

However, CaM is not only a major protein in cytosolic calcium buffering in neurons; it also has a major role in neuronal metabolism, excitability, and intercellular and intracellular signaling. Thus, Aβ(1–42):CaM complexes can also function as intracellular transducers for focalized actions of Aβ peptides, and will be analyzed in the following sections of this review.

## 2. The Roles of CaM in Neurons as Cytosolic Calcium Buffering and Calcium Signaling Molecule—Subcellular Distribution of CaM-Binding Proteins (CaMBPs) in Neurons

Khachaturian [28] proposed the “calcium hypothesis of brain aging and AD”, which defended the idea that sustained changes in calcium homeostasis could be a common pathway for aging and the neuropathological changes associated with AD. Later, calcium dyshomeostasis in AD received further experimental support. For example, Kuchibhotla et al. [29] reported that the resting Ca^2+^ concentrations in the spines and dendrites of pyramidal neurons in the neocortex are higher than normal in neurons located close to amyloid deposits. Similarly, the resting level of Ca^2+^ in cortical neurons of 3xTg-AD animals was 247 nmol/L, which was twice that found in non-Tg controls (110 nmol/L) [30]. These measurements were consistent with many other studies that indicate that Ca^2+^ signaling is up-regulated in AD [31].

Steady resting cytosolic calcium ranges between 70 and 150 nM in different types of neurons in culture, and peaks below 1 μM upon transient plasma membrane depolarization by action potentials or upon stimulation by excitatory neurotransmitters [32,33,34,35,36]. In addition, there are large time-dependent and space-dependent fluctuations in calcium concentrations within different cytosolic regions, soma, and axo-dendritic extensions upon neuronal stimulation. Furthermore, the association with plasma membrane lipid raft nanodomains of voltage-operated calcium channels, NMDA and AMPA-glutamate receptors, and plasma membrane calcium pumps, leads to the generation of high calcium concentration transients near the plasma membrane, both in the soma and synapses [36,37]. Since the dissociation constant of calcium from CaM is relatively high, i.e., between 0.2 and 0.5 μM [23,38], the potency of CaM as a calcium buffer in different cytosolic compartments in neurons is, not only dependent on the local concentration of CaM, but also on the local concentration of free calcium. In addition, fluctuations of cytosolic calcium in neurons strongly shift the equilibrium between the calcium-saturated open conformation of Ca^2+^-CaM (Ca^2+^_4_-CaM) and the close conformation of apo-CaM (minus Ca^2+^) [38]. Although the interaction of Aβ(1–42) with CaM did not significantly alter calcium binding to CaM, the affinity of Aβ(1–42) for apoCaM was found to be approximately 20-fold lower [23].

The CaM conformation changes from a closed to open configuration upon calcium-binding and allows Ca^2+^/CaM to bind target proteins with high affinity (Kd = 10^−7^ to 10^−11^ M) [39,40]. The majority of CaMBPs bind Ca^2+^-CaM, while a small number of proteins, such as neuromodulin and neurogranin, only bind to apo-CaM [41]. 

CaM binds and modulates the activity of the plasma membrane calcium pump (PMCA), a protein that has a recognized major role in the control of the homeostasis of cytosolic calcium in neurons [35,42,43]. In addition, CaMBPs play a major role in neuronal function and excitability, and many of them present significant compartmentation within subcellular neuronal structures. In this review, we will focus on CaMBPs expressed in specific brain areas (hippocampus and cerebral entorhinal, and temporal and frontal cortex), which are prone to degeneration in AD and of which functional impairment has been suggested to underlie the loss of neuronal functions and/or intracellular calcium dysregulation in this disease.

Tau and Aβ peptides are components of neuropathological hallmarks of AD, and tau, amyloid β precursor protein, and β-site APP-cleaving enzyme 1 (BACE1) are CaMBPs [44,45]. In addition, CaM binds and modulates the activity of several protein kinases involved in tau hyperphosphorylation, such as CaMKII, cyclin-dependent kinase 5, and glycogen synthase kinase 3α [26,46]. Tau belongs to the family of microtubule-associated proteins that function in microtubule assembly and stability; tubulin itself and microtubule-associated protein 2 are also CaMBPs [47,48].

CaMKs (CaMKI-IV) are a family of serine/threonine (Ser/Thr) protein kinases [49], which are abundantly expressed in the brain; in some regions, such as the hippocampus, CaMKII levels can reach to 2% of total proteins [50]. Upon activation by Ca^2+^/CaM binding, CaMKs phosphorylate Ser/Thr residues of their target proteins and trigger the activity of different substrates [51]. The multiplicity of substrates is a key feature of multifunctional CaMKs, since this modulates the activity of many neuronal Ca^2+^ effectors that mediate a wide range of neuronal processes that are critical for cognition and many other brain functions. CaMKII is the most studied member of the CaMK family owing to its central role in neuronal plasticity and cognitive functions, such as learning [52]. CaMKII has different properties according to its subunit composition and isoform type (α, β, γ, and δ), because each isoform has different features, such as calcium trapping kinetics, subcellular distribution, and affinity for other proteins [53,54]. The Ca^2+^/CaM complex binds to the regulatory region of CaMKII and produces a conformational change that activates the phosphorylation of its substrates [55] as well as autophosphorylation at Thr286 in the α isoform and Thr287 in the β, ƴ, and δ isoforms [54]. Autophosphorylation prevents the enzyme from reverting to its inactive form and maintains CaMKII activity, even after the intracellular Ca^2+^ levels decrease, which allows for CaMKII-autonomous and Ca^2+^-independent activities [56]. Many studies have shown that α-CaMKII activity is essential for the induction of long-term potentiation (LTP) in hippocampal slices, spatial learning, and memory [57,58,59,60,61,62,63].

Calcineurin (or protein phosphatase 2B) is a Ca^2+^ and calmodulin-dependent Ser/Thr protein phosphatase [64], which is activated by nanomolar concentrations of Ca^2+^ [65]. Calcineurin regulates proteins that play key roles in synaptic transmission and neuronal excitability [66]. Calcineurin is mostly expressed in the cerebral cortex, hippocampus, and striatum, as well as in the cerebellum [67]. In neurons, calcineurin is found in the perikarya and nucleus [67] and in synaptic terminals [68]. Depending on the strength, duration, and site of Ca^2+^ stimulus, calcineurin may either increase or decrease synaptic efficacy and cell excitability through the modulation of ion channels, neurotransmitter receptors, cytoskeletal proteins, kinases, other phosphatases, and transcription factors [69].

Neuronal nitric oxide synthase (nNOS) and adenylyl cyclases 1 and 8 (AC1 and AC8) are other types of Ca^2+^/CaM-stimulated enzymes that generate second intracellular messengers of high relevance for learning and memory [70,71,72]. nNOS is highly expressed in the hippocampus with a widespread intracellular distribution (nucleus, cytoplasm, plasma membrane, and synaptic spines) and mediates synaptic plasticity, including LTP and neuronal survival and signaling [72,73]. AC1 is the only adenylyl cyclase specific to neurons and is expressed in the hippocampus and cerebellum [71,74], while AC8 is expressed mainly in the hippocampus and slightly in the cortex [70,71]. AC1 is directly stimulated by CaM and calcium at a concentration just above that of resting free Ca^2+^ in neurons (150–200 nM) [70,75]. AC8 is also stimulated by CaM, but its Ca^2+^ sensitivity is approximately five-fold lower than that of AC1 [76]. Both ACs are required for some types of synaptic plasticity, including LTP and long-term memory formation (LTM) [70,77]. In addition, overexpression of AC1 in mouse forebrain showed an increase in memory for novel objects and enhanced L-LTP [21,78]; however, as noted above, a sustained dysregulation of calcium homeostasis has been reported in AD [31,41] and it should be noted that Ca^2+^/CaM-dependent phosphodiesterase isoforms modulate intracellular cAMP dynamics in response to elevation of cytosolic Ca^2+^ [79].

Ion channels, such as the small-conductance potassium (SK) channels, KCNQ potassium channels, cyclic nucleotide-gated channels, NMDAR, transient receptor potential channels, ryanodine receptors (RyR), voltage-gated Ca^2+^ channels (VGCCs), and voltage-gated Na channels, are also modulated by CaM [80]. In this work, we will only highlight the modulation of Ca^2+^ channels that are recognized to play a major role in learning and memory, two brain functions that are largely impaired in AD, by CaM.

Calcium influx through VGCCs is crucial for vesicular release of neurotransmitters, intracellular signaling pathways, gene expression, and synaptic plasticity [81]. Among them, L-type VGCC (LTCC) plays an important role in neuronal plasticity, learning and memory, and an alteration in the function and/or regulation of these channels has been associated with neuropsychiatric diseases, migraine headaches, cerebellar ataxia, autism, schizophrenia, bipolar disorder, and depression [82,83]. The isoforms of LTCC, Cav1.2 and Cav1.3, are more highly expressed in the brain, and they have received increased attention regarding their role in neurological and psychiatric diseases [82]. Both Cav1.2 and Cav1.3 can be found in neuronal cell bodies and proximal dendrites in the hippocampus and have been involved in the regulation of many Ca^2+^-dependent functions, e.g., protein phosphorylation, enzyme activity, gene expression, and neurotransmission [84]. Furthermore, calcium influx through LTCC is limited by constitutively bound CaM, which leads to Ca^2+^-dependent inactivation [85] and prevents neuronal damage due to excessive Ca^2+^ entry [86]. Briefly, the CaM conformational change upon Ca^2+^ binding promotes inactivation of LTCC by interaction with additional effector sites of the C-terminal domain in Cav1.2, and, in case of Cav1.3, also of the N-terminal domain [86]. The strength of Ca^2+^-dependent inactivation can be adjusted by regulating the strength of CaM binding by displacement of CaM from its C-terminal interaction sites [86,87].

NMDAR are critical for the expression of LTP in hippocampal and cortical regions [88,89], and overexpression of the NR2B subunit in the hippocampus has been found to increase the amplitude of LTP in the CA1 region and to enhance learning in mice [90]. NMDAR can be directly or indirectly modulated by CaM or CaMKII, respectively [91]. In direct CaM-NMDAR modulation, after calcium entry, CaM induces inactivation of NMDAR through a reduction of its open rate and mean open time by binding to two regions of the C terminal domain of the NR1 subunit, i.e., a high affinity site at the alternatively spliced C1 exon (Kd ≈ 4 nM) and a lower affinity site at the neighboring C0 region (Kd ≈ 87 nM) [92]. This Ca^2+^-dependent inactivation provides an important feedback inhibition of Ca^2+^ influx, preventing excessive Ca^2+^ entry through NMDAR that can lead to neurodegeneration and excitotoxicity [93]. In addition, the indirect modulation of NMDAR by CaMKII is crucial for LTP and long-term depression (LTD) in the brain [94], as disruption of the interaction of NMDAR/CaMKII produces deficits in hippocampal LTP and spatial learning [95]. In the postsynaptic compartment, Ca^2+^ influx through NMDAR activates CaMKII and its translocation from cytosol to postsynaptic density membranes, where it binds to NMDAR subunit 2B (NR2B) [96]. CaMKII/NR2B binding requires an initial Ca^2+^/CaM stimulus, but this interaction persists, even after dissociation of CaM from the complex due to the autophosphorylation of CaMKII at Thr286 [94].

Other neuronal CaMBPs expressed in the hippocampus, such as myosin light chain kinase [97,98], spectrin [99], and fodrin [100], play major roles in the cytoskeleton structure and dynamics, and they also play key roles in neuronal activity and interneuronal connectivity. In addition, Ras-guanine nucleotide-releasing factor 1 (Ras-GRF1), which is also expressed in CA1 neurons of the hippocampus, has been shown to be involved in the induction of LTP and LTD associated with spatial learning and long-term memory [101,102]. Finally, glycogen phosphorylase kinase, in addition to its role in the regulation of glycogenolysis in the brain [103], can also phosphorylate the apo-CaM-binding regulatory regions of neuromodulin (Nm) and neurogranin (Ng) [104], which are neuronal specific proteins that are known to play major roles in neuronal plasticity and LTP (see below).

However, at normal resting cytosolic calcium concentrations close to 100 nM or lower in neurons, CaM is mainly in the apo-CaM conformation. Apo-CaM is largely associated with three proteins in neurons: Nm, Ng, and regulator of CaM signaling (RCS) [21]. Nm, also known as GAP43 or B-50 or P-57, is an abundant presynaptic protein and was the first CaMBP discovered to have a higher affinity for CaM in the absence of Ca^2+^ [21]. Nm accumulates in axonal growth cones and helps their navigation to appropriate target sites during the development of the nervous systems [105]. Furthermore, it is involved in neurite extension and neuronal plasticity, neuroregeneration, regulation of neurotransmitter release at the presynaptic terminal, and in LTP [106]. It has been demonstrated that Nm binds to apo-CaM at the presynaptic membrane and releases it locally under two different mechanisms: (1) when there is an increase in intracellular Ca^2+^ or (2) upon phosphorylation at Ser41 by protein kinase C (PKC), which blocks apo-CaM-binding to Nm [106,107]. 

Ng (also known as RC3, BICKS and P17) is a postsynaptic neuronal-specific CaMBP that is expressed in the cerebral cortex and hippocampus [108]. In neurons, it is expressed in the cytoplasm and in dendritic spines, where it participates in synaptic signaling via regulation of CaM availability [109]. Ng binds to CaM only in calcium-free medium, suggesting that Ng could serve as a reservoir for apo-CaM and as a Ca^2+^ sensor [110]. An IQ motif of Ng mediates its interaction with apo-CaM and with phosphatidic acid, and phosphorylation by PKC at Ser36 blocks the binding of Ng with apo-CaM or phosphatidic acid [111]. Ng-knockout mice display an apparently normal phenotype, but show severe deficits in spatial and emotional learning and a decrease in LTP induction [111] as well as a slightly enhanced LTD [112]. Indeed, Ng mutants lacking the ability to bind to apo-CaM are unable to potentiate synaptic transmissions [110]. In conclusion, similar to Nm, Ng has an important role in the neuroplasticity mechanism of learning and memory [113].

RCS, also known as ARPP-21 or cAMP-regulated phosphoprotein 21kDa, is a PKA-regulated phosphoprotein expressed in brain regions receiving dopaminergic innervation [114]. RCS is enriched in *caudate-putamen*, *substantia nigra*, *nucleus accumbens* and olfactory tubercle, but also displays intermediate levels of expression in the cerebral cortex and hippocampus [115]. The G protein-coupled receptor (GPCR)-dependent activation of protein kinase A (PKA) leads to phosphorylation of RCS at Ser55, and increases its binding to CaM [116], preventing CaM from binding to CaM-regulated phosphatase calcineurin [21].

## 3. Modulation by Aβ of CaMBPs, Which Play Major Roles in Aβ Production in Neuronal Calcium Homeostasis and LTP

### 3.1. The Relevance of Aβ:CaM Complexation for the Regulation of Neurotoxic Aβ Oligomer Formation

Clinically, AD is divided into sporadic AD (sAD) and familial AD (fAD). The fundamental role of Aβ in AD is derived from studies of fAD, which accounts for 1–5% of patients with AD [117], who have autosomal dominant mutations or duplications in the amyloid precursor protein (APP) or mutations in the presenilin-1 (PSEN1) and presenilin-2 (PSEN2) genes [118,119]. These mutations result from changes in APP proteolysis by the γ-secretase complex, leading to an increase in the formation of toxic Aβ(42/40) oligomers, which induce synapse loss and neuronal toxicity [120,121]. As fAD is pathologically similar to sAD, with the difference being that fAD generally has an early onset and the symptoms progress more rapidly, it is believed that Aβ(1–42) over-production is also a main factor in sAD [121]. PSEN mutations contributes to over 90% of fAD cases, and several studies have shown that intracellular calcium dysregulation due to these mutations takes place before the formation of Aβ plaques and neurofibrillary tangles (NFT) in AD brains, highlighting that modifications in cytoplasmic calcium may be an early event at the onset of AD [122,123]. The increase of cytosolic calcium, in turn, leads to a CaM-mediated stimulation of the amyloidogenic protease, BACE1. In vitro experiments have shown that this calcium–CaM dependent stimulation of BACE1 is about 2.5-fold [45]. Moreover, Giliberto et al. [124] showed that the treatment of neuronal and neuroblastoma cells with 1 μM soluble Aβ(1–42) increased BACE1 transcription and that this was reverted by an anti-Aβ(1–42) antibody. It has been suggested that this could be due to Aβ-induced oxidative stress, because this increase in BACE1 transcription was shown to be mediated by the activity of NFκB [125]. Furthermore, an up-regulation of BACE1 expression in several vascular risk factors for AD development, including hypoxia, hyperglycemia and hypercholesterolemia, has been shown and was reviewed in [126]. Therefore, free Aβ generates a positive feedback loop of Aβ production, and this is likely to play a major role in brain degeneration, both in fAD and sAD. On these grounds, lowering of free Aβ by complexation with CaM can be seen as a cellular defense response to slow down the formation of neurotoxic Aβ in neurons. In addition, the increase of Aβ(1–42):CaM complexes elicited by the rise in Aβ production reduces the availability of free CaM for stimulation of BACE1 activity, providing feedback inhibition of amyloidogenic Aβ production. Of note, it has been shown that the CaM antagonist W7 stimulates cleavage of APP through a non-amyloidogenic pathway [127,128]. To the best of our knowledge, the possibility that Aβ(1–42):CaM complexes may also inhibit BACE1 activity has not yet been experimentally assessed.

### 3.2. The Relevance of Aβ:CaM Complexation for the Alteration of Intracellular Calcium Homeostasis Induced by Aβ

PSENs modulate intracellular Ca^2+^ homeostasis through direct interaction with three components of the endoplasmic reticulum (ER), namely, inositol triphosphate receptors (IP3R), RyR, and sarco(endo)plasmic reticulum Ca^2+^-ATPase (SERCA) [129]. Mutations of the PSEN2 gene enhanced Ca^2+^ release through IP3R [130] and mutations in PSENs can also modulate capacitative calcium entry, a refilling mechanism for depleted Ca^2+^ stores [122,131,132]. Store-operated calcium entry (SOCE) disruption is consistently observed in AD and is manifested as attenuated Ca^2+^ entry in the primary neurons of AD mice with human mutant PSEN1 knocked in, or in skin fibroblasts from familial AD patients [133]. In addition, it has been reported that STIM2 expression levels are down-regulated by fAD-linked PS1 mutations and, thus, insufficient signal is transferred to the plasma membrane to activate SOCE when ER Ca^2+^ is depleted [134]. In addition, PSEN also acts as an ER Ca^2+^ leak channel and fAD mutations in PSEN1 disrupt this function [135,136], leading to overloaded ER Ca^2+^ stores and increased ER Ca^2+^ release in PSEN double knockout fibroblasts and in fibroblasts transfected with mutant PSEN1 and PSEN2 constructs [121]. Additionally, in fAD, PSEN mutations increase RyR-mediated Ca^2+^ release, either due to enhanced expression of channel proteins or sensitization of the channel activity through PSEN–RyR protein interactions [135,137]. In addition, apoE4, a genetic risk factor for AD, may also amplify ER Ca^2+^ release through RyR, thereby stimulating the formation of Aβ plaques and neurofibrillary tangles [138,139]. In vitro experiments have shown that application of soluble Aβ oligomers causes a large increase in RyR activity due to an approximately 10-fold increase in the channel open probability [140] and stimulates RyR-mediated Ca^2+^ release in hippocampal neurons in culture [141]. It must be noted that the increase of the open channel activity of RyR was measured with the application of micromolar concentrations of Aβ(1–42) to skeletal muscle fibers. Thus, it seems that oligomeric Aβ may only further potentiate excessive ER Ca^2+^ release by direct interaction with RyR at concentrations that are not reached within the neurons at the early stage of AD brain degeneration. However, indirect modulation by Aβ-induced oxidative stress may underlie the Aβ-induced activation of RyR observed in hippocampal neurons in culture after 2–3 h of incubation with concentrations of Aβ(1–42) oligomers ≥500 nM [141]. Therefore, sequestration of Aβ oligomers by CaM could be expected to protect against the increase of ER Ca^2+^ release through RyR. In addition, it has been reported that mutation or deletion of PSEN alters the ER calcium refilling process through the SERCA pump and may contribute to the pathogenesis of AD [142]. Indeed, it has been recently shown that increasing SERCA activity helps to maintain ER calcium and improves memory and cognition in APP/PSEN1 mice, as SERCA activation can sequester more cytosolic Ca^2+^ and prevent the apoptosis induced by mitochondrial signaling [143].

While ER calcium release stimulates Aβ production (see above), the produced Aβ can inhibit the activity of plasma membrane Ca^2+^ extrusion systems, PMCA, and sodium-calcium exchanger (NCX). According to Mata and colleagues, PMCA is the only Ca^2+^ pump in which Ca^2+^ dependence activity is altered in membranes of AD brains compared to control brains and, also, is the only pump in the brain which is directly inhibited by Aβ [144,145]. Mechanistic studies indicated that Aβ aggregates are more potent inhibitors of PMCA activity than monomers [145] and the Aβ inhibitory effect is due to the interaction of Aβ with the C-terminal tail of PMCA [43]. In addition, the inhibitory effect of Aβ could be blocked by pretreating the purified protein with Ca^2+^/CaM, the main endogenous activator of PMCA [145]. Additionally, Aβ can inhibit NCX activity, either by direct interaction with the hydrophobic surface of NCX and/or with the lipid bilayer of the plasma membrane [146]. It has been shown that 1 μM Aβ(1–40) stimulates NCX activity three-fold in the reverse mode in human astrocyte-derived glioblastoma cells with a time delay of 400–500 seconds after application of this peptide [147]. The short time for NCX activity modulation by Aβ(1–40) suggests that this may be due to the direct interaction between this peptide and NCX; however, a titration with submicromolar concentrations of Aβ(1–40) was not reported by these authors and data are not available to obtain a dissociation constant of this peptide from NCX. Of note, impaired hippocampal LTP and memory-related behaviors have been recently reported for NCX2+/− or NCX3+/− mice [148]. The synaptosomal expression of NCX1, NCX2, and NCX3, the three variants of NCX, were investigated in AD parietal cortex by Sokolow et al. [149]. These authors found that NCX2-positive terminals were increased in the AD cohort, while NCX3-positive terminals were reduced, and they demonstrated that the three isoforms co-localized with Aβ in synaptic terminals [149]. This co-localization could increase the local concentration of Aβ near NCX, and this would favor the modulation of NCX activity by its direct interaction with Aβ peptides at lower cytoplasmic free Aβ concentrations. If this is the case, complexation of Aβ with CaM should antagonize NCX modulation by Aβ peptides, protecting against the rise of cytosolic calcium by calcium entry through NCX. Although these authors also reported that all three variants are up-regulated in nerve terminals containing Aβ [149], a recent study carried out in APP23 and APP-KI transgenic mice demonstrated that both protein and mRNA levels of NCX2 and NCX3 isoforms were down-regulated in hippocampal CA1 neurons [148]. These findings suggest that further studies are required to unveil the functional modifications and expression patterns of all NCX isoforms and their roles in AD pathology.

Since Aβ can inhibit the major Ca^2+^ extrusion systems of the neuronal plasma membrane, it can be foreseen that an increased ER Ca^2+^ release, induced by mutations in PSEN proteins, should eventually lead to mitochondrial Ca^2+^ overload and apoptotic pathways. Indeed, this is an effect of exogenous Aβ peptides that has been experimentally demonstrated [150,151]. Cumulative lines of evidence have demonstrated that mitochondrial Ca^2+^ signaling is altered in AD due to mutations in the PSEN proteins [152,153]. The excess in cytosolic Ca^2+^ caused by enhanced ER Ca^2+^ release caused by mutant PSENs is, at least in part, counterbalanced by the Ca^2+^ uptake through the voltage-dependent anion-selective channel protein and the calcium uniporter of the mitochondria. A sustained increase in mitochondrial Ca^2+^ concentration impairs ATP production, increases reactive oxygen species (ROS) production, and the opening of the mitochondrial permeability transition pore [154]. Several studies have proposed that enhanced neuronal apoptosis and increased ROS production are major factors in the neurodegeneration observed in AD, and the accumulation of mitochondrial Ca^2+^ has been shown to be significantly implicated in these neurotoxic pathways [153,154]. Upregulation of genes related to mitochondrial energy metabolism and apoptosis has been already reported in an AD transgenic mouse model overexpressing a mutant form of APP at different stages of AD progression [155]. This upregulation is likely an adaptive response to restore the mitochondrial functions impaired by the sustained increase in cytosolic Ca^2+^ induced by Aβ. However, it seems that this is not enough to protects against the loss of mitochondrial function produced by the oxidative damage associated with the overproduction of ROS induced by mutant APP and soluble Aβ [152]. Indeed, Wang et al. [156,157] demonstrated that essential proteins for mitochondria fission and fusion, which are needed to maintain synaptic activity in healthy neurons, are altered by enhanced Aβ production when APP is overexpressed in human neuroblastoma cell line M17, and, also, by treatment with oligomeric Aβ(1–42). Furthermore, these authors showed that altered levels of mitochondrial fission/fusion proteins in M17 cells, and in differentiated hippocampal neurons mimicking changes observed in AD neurons, led to an increase in mitochondrial fragmentation and abnormal distribution, which contribute to mitochondrial and neuronal dysfunction [156,157]. Later, Silva-Álvarez et al. [158] showed that treatment of hippocampal neurons in culture with a concentration of 500 nM Aβ(1–42) oligomers for 2–5 hours elicits a large loss of mitochondria per neuron with respect to control neurons. In addition, it was shown by Area-Gomez et al. [159] that the contact sites between mitochondria and ER are rich in PSEN, and it was later demonstrated that mutations in PSEN and APP can upregulate mitochondria-associated ER membrane (MAM) functions and generate a substantial increase in ER–mitochondrial connectivity [160]. Since these authors observed the same upregulation in MAM function and ER mitochondrial communication in fibroblasts from patients with sAD, without mutations in PSEN1, PSEN2, and APP, they suggested that MAM-upregulated function is a common feature in both fAD and sAD, and proposed that it may represent a pathogenic initiator of AD [160,161]. In addition, other recent studies have shown that an increase in Aβ production may perturb mitochondria and mitochondria–ER contact site functions, mediating the neurodegeneration observed in AD, see, e.g., [153,162].

### 3.3. The Relevance of Aβ:CaM Complexation for Dysregulation of Calcium-Dependent Neuronal Activity and Excitability Induced by Aβ

The direct modulation of CaMKII by Aβ has a strong impact on neuronal activity and excitability. It has been shown that treating hippocampal neurons with Aβ oligomers impairs αCaMKII activation [163,164] and that Aβ prevents the activation of CaMKII during hippocampal LTP [165]. The inhibition of CaMKII by Aβ may be primarily a neuronal defense mechanism because APP can be phosphorylated in vitro by several kinases, including CaMKII [166], and CaMKII is also a tau kinase, which has been suggested to act in priming tau phosphorylation by cyclin-dependent kinase 5 and glycogen synthase kinase β [167,168]. It should be observed here that Aβ(1–42) has been reported to bind to glycogen synthase kinases 3α and 3β with high affinity [26]. Indeed, the reported dissociation constants of Aβ(1–42) from the glycogen synthase kinase 3α isoenzyme [26] and from CaM [23] are almost identical, ≈1 nM. Furthermore, Dunning et al. [26] demonstrated that binding of Aβ(1–42) to glycogen synthase kinase 3α stimulates hyperphosphorylation of tau. In addition, glycogen synthase kinase 3α has been proposed to enhance Aβ production through γ-secretase stimulation [169]. Therefore, the inhibition of CaMKII by Aβ may counteract, at least in part, the stimulation of glycogen synthase kinase 3-dependent tau phosphorylation by nanomolar Aβ concentrations. Moreover, it has been proposed that, outside of synapses, αCaMKII is hyperactive and could contribute to NFT formation since it co-localizes with NFT in the AD brain [164]. To the best of our knowledge, the possibility that CaMKII could also bind Aβ:CaM complexes has not been experimentally assessed, nor has the putative role of these complexes in Aβ-induced CaMKII inhibition.

Inhibition of CaMKII prevents against the phosphorylation of nNOS at Ser847, which inhibits the activation of nNOS by Ca^2+^/CaM [170,171]. This is a neuroprotective effect of CaMKII inhibition by Aβ, because nitric oxide-induced inhibition of NMDAR protects neurons against the toxicity elicited by the excessive increase in cytosolic Ca^2+^ produced by sustained stimulation of these glutamatergic receptors [172]. It has been shown that nNOS can be inhibited by several Aβ peptides with inhibitor constants ranging from 0.81 to 14 μM [173]; however, these high concentrations of Aβ peptides are unlikely to be reached within the neurons until a late stage of AD brain degeneration.

In vivo, the activation of CaMKII is under the negative control of calcineurin-dependent phosphatase activity [174,175] and is essential for LTP generation [57,176]. Calcineurin-dependent subcellular relocation of autophosphorylated αCaMKII also occurs in Aβ oligomer-treated primary neuronal cultures [177,178]. A shift of p(T286)-αCaMKII from apical dendrites/spines to the soma of CA3 pyramidal neurons, which is blocked by inhibition of the phosphatase calcineurin, is also found in a mouse model of sAD, in which amyloid oligomers are injected into the ventricles [178]. Post-mortem analyses and studies with AD models indicate that T286-autophosphorylation of αCaMKII is decreased at the synapses in the disease [164]. It should be recalled that this autophosphorylation is essential for NMDAR-dependent LTP at CA1 synapses and for spatial memory formation [179,180]. Indeed, knockdown of CaMKII mimics the reduced surface expression of AMPA receptor subunit GluA1 and decreased AMPA receptor-mediated synaptic transmission, which is reversed by CaMKII overexpression [177]. An analogous observation is seen when treating rat hippocampal slices with Aβ(1–42). In this experimental model, Aβ inhibits CaMKII activation and blocks the stimulation-dependent phosphorylation of a CaMKII-specific site on GluA1 [165]. Moreover, treatment that enhances CaMKII activity also improves long-term memory in a mouse model of AD [181].

Acute application of synthetic Aβ elicits inhibition of LTP in area CA1, or *dentate gyrus*, of rat hippocampus [182,183,184,185], as well as in conditioned culture medium containing Aβ species secreted by cells transfected with human APP [9]. In the *dentate gyrus* Aβ inhibition of LTP was blocked by specific inhibitors for calcineurin, indicating that increased calcineurin activity contributes to Aβ-induced LTP inhibition [183]. Thus, Aβ can also alter LTP by disrupting the dynamic balance between protein phosphorylation and dephosphorylation of CaMKII. It has been proposed that the increase in cytosolic calcium induced by Aβ triggers calcineurin hyperactivity [186]; however, the possibility that Aβ and/or Aβ:CaM complexes might bind to calcineurin does not seem to have been explored until now. At least, this is a priori relevant for the dynamics of tau:calcineurin complexes and ultimately for the modulation of the extent of tau phosphorylation, since binding of Ca^2+^-CaM to calcineurin disrupts its interaction with tau and lowers its ability to dephosphorylate tau [187]. In addition, despite the fact that extracellular Aβ applied to hippocampal slices preferentially targets synapses [13], experimental data are lacking to exclude that Aβ could alter the association/dissociation kinetics of apo-CaM with CaM reservoir proteins, such as Nm, Ng, or RCS, in synaptic terminals.

Accumulated evidence has demonstrated that soluble Aβ oligomers bind to NMDAR and induce Ca^2+^ influx [187], which leads to further Ca^2+^ release into the cytosol from internal stores within the spine [188], the major component of spine calcium transients. In addition, it has been demonstrated that Aβ oligomers associate and co-localize with dendritic trees [189], which are postsynaptic membranes enriched in NMDAR [190]. A direct activation of NMDAR by Aβ(1–42) oligomers has been demonstrated with NR1/NR2A and NR1/NR2B receptors, heterologously expressed in *Xenopus laevis* oocytes [191], but the binding sites of Aβ oligomers in NMDAR subunits remain to be identified. This elicits a pathological level of Ca^2+^ signaling, producing a gradual loss of synaptic function and, ultimately, neuronal excitotoxic cell death. However, the molecular mechanisms underlying the activation of NMDAR in the AD brain remain controversial, because NMDAR are activated by oxidative stress [192] and Aβ oligomers also induce an increase in cellular oxidative stress [187,193]. On the other hand, studies with astroglial and neuronal cells show that Aβ impairs glutamate uptake/recycling mechanisms, contributing to AD-associated excitotoxicity and neurodegeneration [194,195]. Interestingly, hippocampal neurons are more susceptible to this type of injury than cortical neurons, and in organotypic hippocampal slices, CA1 neurons show greater susceptibility than CA3 or *dentate gyrus* neurons [164]. This reflects the hierarchical decline of brain areas during disease progression [196]. This provides rationale for the clinical trial of memantine, the NMDAR antagonist, as a neuroprotective treatment for AD [197].

LTCC have long been implicated in aging and AD [198]. A decrease in LTCC activity has been reported in the hippocampus of APP/PS1 double-transgenic mice [199]. In a recent study, Poejo et al. [24] reported the inhibition of L-type calcium channels of cerebellar granule neurons by submicromolar cytosolic concentrations of Aβ(1–42) dimers, likely mediated by Aβ-CaM complexes. Owing to the high contribution of calcium influx through LTCC to increasing the resting cytosolic calcium in neurons [36,200,201,202], the inhibition of LTCC by Aβ seems another compensatory neuroprotection mechanism to prevent pathogenic cytosolic Ca^2+^ dysregulation. Adaptive control of the activity of LTCC upon exposure to Aβ is also suggested by experiments with astrocytes cultures, since acute exposure of astrocytes to murine Aβ(1–42) increased the expression of the Cav1.2 α1-subunit, whereas chronic treatment decreased it, showing that Aβ can differentially regulate LTCC expression, depending on the incubation time [203]. However, the molecular mechanism(s) accounting for the inhibition of LTCC by Aβ found in primary cultures of cerebellar granule neurons remains to be settled, as LTCC are activated by CaMK-dependent phosphorylation but are inactivated by CaM binding [204,205]. Noteworthy, nimodipine, a dihydropyridine derivative and LTCC antagonist, has beneficial effects in AD patients and slows the progression of the disease [206]. Although two large-population, long-term cohort studies have proved the protective role of Ca^2+^ channel blockers over other types of antihypertensive drugs on the risk of dementia among elderly hypertensive populations [207,208], the clinical effects of each specific LTCC blocker remain controversial [209].

## 4. Conclusions

The high affinity of small Aβ oligomers for CaM and the high concentration of CaM in neurons reveal a major role of CaM for Aβ buffering in neurons, which protects against the rise of free concentrations of neurotoxic Aβ peptides. In turn, the concentration of Aβ-CaM complexes within neurons increases as a function of time after induction of Aβ production, and free Aβ will rise sharply when accumulated Aβ exceeds all available CaM, i.e., when it reaches total micromolar Aβ. Intraneuronal Aβ oligomers found in the AD brain can arise from endogenous Aβ peptide production as well as from vicinal reactive glial cells [210], as inflammation is now recognized to foster AD brain degeneration. The slow kinetics of Aβ uptake and its internalization by neurons [12,211] is likely one of the factors that slows down the time course of the neurotoxicity of exogenous Aβ, and is relevant for the comparison and integration of the results and conclusions of studies performed in cell cultures after exposure to exogenous Aβ. In addition, lipid rafts have an important function in Aβ uptake and its internalization in neurons [20,212,213], and associated with them are calcium entry and extrusion systems that control resting cytosolic calcium homeostasis and neuronal excitability, such as PMCA, NCX, LTCC and NMDAR [36,37]. Thus, lipid rafts provide a physical link between the known alteration of cholesterol metabolism and dysregulation of cytosolic calcium in AD. On these grounds, it is to be expected that the Aβ-triggering of molecular mechanisms for the onset of neuronal cytosolic calcium dysregulation will be different for endogenously generated Aβ in the early stages of fAD and for the Aβ produced by vicinal reactive glial cells, probably a major source of Aβ during AD brain degeneration. 

In addition, Aβ-CaM complexation is likely to play a major role in the functional regulation of CaMBPs by Aβ, either in sensitivity or activity modulation. This has been largely overlooked until now, and it may have relevant implications for neuronal Aβ production, since APP and BACE1 are CaMBPs, for tau phosphorylation and for neuronal calcium dysregulation in AD, which mediates loss-of-function and neurodegeneration in AD brains. The identification of the primary target proteins for non-endogenous intraneuronal Aβ, of which functional impairment initiates cytosolic calcium homeostasis dysregulation as well as the critical point of no return, are still pending issues due to the following major reasons: (1) a lack of assessment of total intracellular Aβ concentrations in experiments with cell cultures reporting cytosolic calcium dysregulation; (2) a lack of the dissociation constant of the direct interaction between Aβ and Aβ-CaM complexes with target proteins; and (3) a lack of measurements of the putative oxidative modifications of calcium channels and pumps in cell cultures after different times of exposure to exogenous Aβ. It should be recalled that that Aβ stimulates intracellular ROS production [190] and that the calcium transport systems that are more relevant for the control of intracellular calcium homeostasis are highly sensitive to a sustained cellular oxidative stress [36,214,215]. However, the experimental data accumulated so far allow us to envisage cellular adaptive responses, i.e., up-regulation and down-regulation of gene and protein expression levels, to compensate for the alteration of intracellular calcium homeostasis upon acute and chronic exposure of neurons both in vitro (cell culture) and in vivo (animal models) to Aβ stress.

## Abbreviations

Aβ	Amyloid β peptide
AC	Adenylate cyclase
AD	Alzheimer’s disease
AMPA	α-Amino-3-hydroxy-5-methyl-4-isoxazolepropionic acid
APP	Amyloid precursor protein
BACE1	β-Site APP-cleaving enzyme 1
CaM	Calmodulin
CaMBPs	Calmodulin binding proteins
CaMKs	Ca^2+^/calmodulin-dependent protein kinases
ER	Endoplasmic reticulum
fAD	Familial or hereditary Alzheimer’s disease
IP_3_R	Inositol trisphosphate receptor
Kd	Dissociation constant
LRP1	Low density lipoprotein receptor-related protein-1
LTCC	L-type calcium channels
LTD	Long-term depression
LTM	Long-term memory
LTP	Long-term potentiation
MAMs	Mitochondria associated ER membranes
NCX	Sodium-calcium exchanger
NFT	Neurofibrillary tangles
Ng	Neurogranin
Nm	Neuromodulin
NMDA	N-methyl D-aspartate
NMDAR	N-methyl D-aspartate receptors
nNOS	Neuronal isoform of nitric oxide synthase
PKA	protein kinase A
PKC	Protein kinase C
PMCA	Plasma membrane calcium pumps
PSEN	Presenilin
RCS	Regulator of calcium signaling
RyR	Ryanodine receptors
ROS	Reactive oxygen species
sAD	Sporadic Alzheimer’s disease
SERCA	Sarco(endo)plasmic Ca^2+^-ATPase
SOCE	Store-operated calcium entry
VGCCs	Voltage-gated calcium channels
